# Automatic Prostate Gleason Grading Using Pyramid Semantic Parsing Network in Digital Histopathology

**DOI:** 10.3389/fonc.2022.772403

**Published:** 2022-04-08

**Authors:** Yali Qiu, Yujin Hu, Peiyao Kong, Hai Xie, Xiaoliu Zhang, Jiuwen Cao, Tianfu Wang, Baiying Lei

**Affiliations:** ^1^ School of Biomedical Engineering, Health Science Center, Shenzhen University, National-Regional Key Technology Engineering Laboratory for Medical Ultrasound, Guangdong Key Laboratory for Biomedical Measurements and Ultrasound Imaging, Shenzhen, China; ^2^ Key Lab for Internet of Things (IOT) and Information Fusion Technology of Zhejiang, Hangzhou Dianzi University, Hangzhou, China

**Keywords:** prostate, gleason grading, histopathology, PSPNet, prostate - pathology

## Abstract

**Purpose:**

Prostate biopsy histopathology and immunohistochemistry are important in the differential diagnosis of the disease and can be used to assess the degree of prostate cancer differentiation. Today, prostate biopsy is increasing the demand for experienced uropathologists, which puts a lot of pressure on pathologists. In addition, the grades of different observations had an indicating effect on the treatment of the patients with cancer, but the grades were highly changeable, and excessive treatment and insufficient treatment often occurred. To alleviate these problems, an artificial intelligence system with clinically acceptable prostate cancer detection and Gleason grade accuracy was developed.

**Methods:**

Deep learning algorithms have been proved to outperform other algorithms in the analysis of large data and show great potential with respect to the analysis of pathological sections. Inspired by the classical semantic segmentation network, we propose a pyramid semantic parsing network (PSPNet) for automatic prostate Gleason grading. To boost the segmentation performance, we get an auxiliary prediction output, which is mainly the optimization of auxiliary objective function in the process of network training. The network not only includes effective global prior representations but also achieves good results in tissue micro-array (TMA) image segmentation.

**Results:**

Our method is validated using 321 biopsies from the Vancouver Prostate Centre and ranks the first on the MICCAI 2019 prostate segmentation and classification benchmark and the Vancouver Prostate Centre data. To prove the reliability of the proposed method, we also conduct an experiment to test the consistency with the diagnosis of pathologists. It demonstrates that the well-designed method in our study can achieve good results. The experiment also focused on the distinction between high-risk cancer (Gleason pattern 4, 5) and low-risk cancer (Gleason pattern 3). Our proposed method also achieves the best performance with respect to various evaluation metrics for distinguishing benign from malignant.

**Availability:**

The Python source code of the proposed method is publicly available at https://github.com/hubutui/Gleason. All implementation details are presented in this paper.

**Conclusion:**

These works prove that the Gleason grading results obtained from our method are effective and accurate.

## Introduction

The incidence and mortality of prostate cancer have been increasing over the past decades (1). With the high risk of overdiagnosis and overtreatment, there is an urgent need to accurately assess patient prognosis (2–4). Currently, the effective diagnostic index of histopathological biopsy of prostate cancer is still the Gleason grade (5). The Gleason grading system for prostate cancer refers to observing and scoring the cancer cells according to the similarity between the normal tissue and cancer cells (6–9). Pathologists recognize that the prognosis of prostate cancer is between its primary structure and secondary structure (10–12). In 2016, pathologists updated the grading system and redefined the grading criteria 1–5 (13). Although its clinical value has been widely recognized, the grading system is very complex and highly subjective. Moreover, the number of the qualified pathologists is insufficient to meet the global demand for pathological detection of prostate cancer. Therefore, how to use the Gleason grading system effectively to realize early automatic diagnosis and treatment has become an important research topic (14, 15).

Automatic segmentation has the potential to decrease lag time between diagnostic tests and treatment by providing a strong and standardized report of tissue location in a fraction of the time, which would take a pathologist to do so (16). However, the diagnostic process highly relies on the pathologist’s rich personal experience, which is not only time-consuming and labor-intensive but also suffering from high subjective errors (17, 18). Moreover, the diagnostic process is also affected by high interobserver variability from different pathologists and limits its effect on individual patients. In order to solve the above problems, doctors often use computer science and technology to assist diagnosis. Among them, the deep learning method has been successfully introduced into the field of medical image analysis (19–21).

Deep learning algorithms have shown their potential for pathological diagnosis at the expert level in other tasks, such as diagnosing skin tissue lesions and identifying breast cancer metastasis. Long et al. (22) proposed a full convolutional network (FCN), which created a new chapter of semantic segmentation and improved the generalization of dynamic objects with an end-to-end way. Liu et al. (23) proved that FCN with the global average pooling module can enhance the segmentation performance. Subsequently, Noh et al. (24) proposed a coarse-to-fine deconvolution network structure for image segmentation. However, FCN only forecasts on a single scale, which cannot effectively deal with the change of size. Due to the significant variations in appearances of prostate cancers, the automatic grading of prostate tissue segmentation is tedious and troublesome. As histopathology image segmentation and classification images are usually of high resolution, it is challenging for the deep learning model to train and learn discriminative features due to the limited computing resources (25, 26). The blurred boundaries of some histopathological tissue may make this task more challenging.

Apart from the existing problems, there are three main challenges in extracting information from digital histopathology of prostate cancer. The first challenge is the high heterogeneity of cells and tissues. The grading of pathological images is complex. It may have several grades in a histopathology image. The second challenge is high interobserver variability. Under the huge data requirements, experts may have different opinions on the annotation of pictures. The third challenge is to learn features from high-resolution images. Considering the high heterogeneity of data and the difference of expert annotation, it is difficult to extract features in network training. Thus, how to efficiently extract useful features is quite critical.

To address the above challenges, we propose a new system to automatically identify prostate histopathology images in this article. In order to obtain data annotation with high interpretability and good robustness, we use the Simultaneous Truth and Performance Level Estimation algorithm (STAPLE) (27) to synthesize different expert annotations. The STAPLE algorithm can alleviate the heavy task of experts and deal with different expert annotations. The proposed system has the potential to improve prostate cancer prognostics and achieves a high agreement with the reference standard. The established benchmark can assess and compare the state of image analysis and machine learning-based algorithms. It will also help assess the robustness and accuracy of these computerized methods against the suggestion of numerous experts. Given the importance of prostate cancer and challenges of the Gleason grade system to detect and diagnose prostate cancer, the promising results can be quite beneficial in the medical community.

Our main contributions are three-fold:

1. A systematic framework is presented, which is beneficial for providing pathologists with an adequate and effective alternative prostate Gleason grading system.
2. An effective feature extraction strategy is proposed based on the pyramid semantic parsing network, which can extract more effective information and improve the accuracy of disease diagnosis.
3. Our method is validated *via* the Vancouver Prostate Centre and ranks first on the MICCAI 2019 prostate segmentation benchmark, which is consistent with the diagnosis of pathologists.

## Methodology

### Overview of the Proposed Method

In this paper, we utilize the pyramid semantic parsing network (PSPNet) to extract features and then refine those features from different scales *via* the pyramid pooling module. To boost the segmentation performance, we get an auxiliary prediction output, which is mainly the optimization of auxiliary objective function in the process of network training. The network not only includes effective global prior representations but also achieves good results in tissue micro-array (TMA) image segmentation.

### STAPLE Algorithm

**Figure 1:**
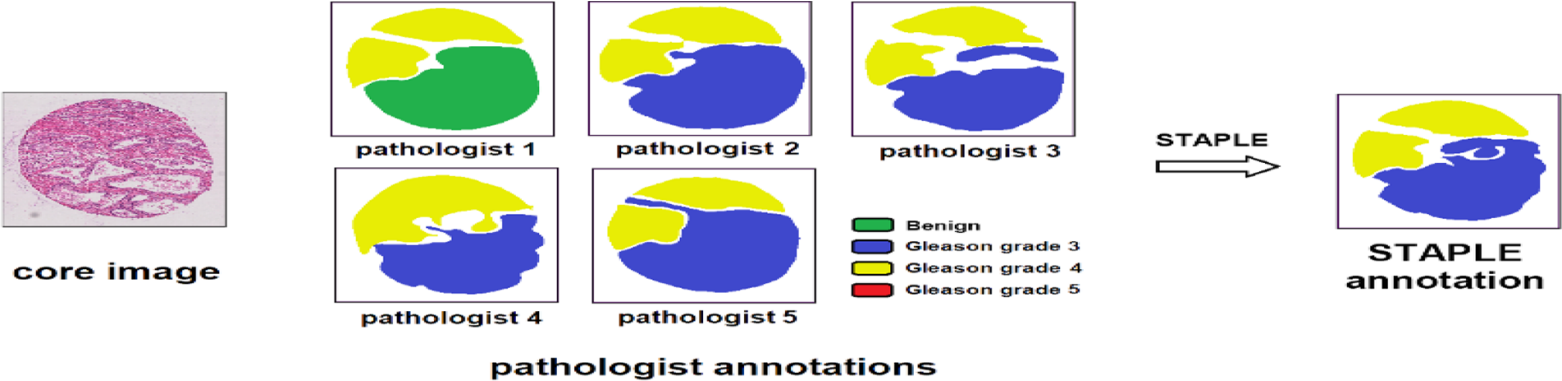
Image processing of pathologist annotations of TMA core images. The STAPLE algorithm, the same as pixel-wise majority voting, fuses the annotations of different pathologists to build the “ground truth label”.

For different segmentation tasks, the expert segmentation is achieved independently, but with the same real segmentation goal. The STAPLE algorithm is used to compare the differences of the final segmentation results based on a rapid interactive level set and hand contours for tumor segmentation (28). The algorithm segments images and calculates the probability of true segmentation simultaneously. The STAPLE algorithm not only takes the systematic deviation caused by the difference of different experts’ annotations into account but also evaluates the annotation quality of each expert. The algorithm can balance the two aspects well and then generate a fuzzy real annotation. We can extract more critical information with different weights of each part of the input, which makes the model to have more accurate judgments and reduces the calculation and storage of the model. The flowchart of the detailed annotation of TMA core images provided by different pathologists is shown in **Figure 1**.

The output of the STAPLE algorithm is a picture with floating point values from 0 to 1, which represents the probability that the pixel point belongs to a specific segmentation target. The picture has the same size as the original image. Further, we want to extend it to multi-category situations. If each pixel has the maximum probability of belonging to a different category, the category label of the point is uncertain. At this point, we take the label of the most experienced expert as the true category label of the point. After preprocessing, the merged labels can be used for subsequent network training and verification.

### Feature Extraction

Feature extraction is a key step in the field of computer vision and image processing, which can be used to extract image information and determine whether the points of each image belong to the useful features. The purpose of feature extraction is to divide the points on the image into different subsets, which are often represented as isolated points, continuous curves, or continuous regions. The quality of extracted features has a crucial impact on the performance of the training network (29).

In recent years, the way of feature extraction has developed from manual design to automatic extraction by the convolution neural network. Since AlexNet was proposed, researchers have devised a variety of convolutional neural network architectures to achieve automatic learning and extraction of features, such as VGG network (30), residual network (ResNet) (31), and densely connected network (DenseNet) (32). Different from classic convolutional neural networks, FCN can handle original input images from an arbitrary dimension and reserve spatial information. It classifies the original image pixel by pixel from the upper sampling and ignores the adjacent information when unpooling the low-resolution feature images. However, the main problem of FCN is that it cannot make good use of category information from a global scene. For the image classification task, the increase in network depth may bring additional optimization difficulties.

The residual block can solve this problem by using the long skip connection in each block and bring good performance. In the deep residual network, the latter layer mainly learns the residual thrown from the previous layer. Influenced by the most widely residual network, our research also uses ResNet as the skeleton network for feature extraction. ResNet is mainly composed of residual modules. Unlike the direct use of convolutional stacking, the residual module introduces residual learning, and hence the network can be made deeper and have more powerful capabilities of feature extraction. The skeleton network can effectively extract the tissue structure features using the prostate cancer pathological slice images and prepare for the next step of feature learning.

### Pyramid Pooling Module

Global pooling has been widely used in the classical complex digital tissue pathological sections to get global image-level features (33). The pyramid pooling module (PPM) is a comparatively good way to fully utilize global information. To reduce the impact of the loss of contextual information between different subregions in the stage of training, some researchers proposed a hierarchical global prior, which contains variations among different subregions and numerous information with different scales (34, 35). Different levels of feature mapping created by the pyramid pool module are ultimately flattened and then connected to the full connection layer as input. We can utilize the global prior technique to eliminate the negative effects of fixed size constraints for the training of the convolutional network (36). The PPM collects different scales of information, which is more typical than global pooling (37). This multi-scale pooling can intuitively maintain global context information and global information of different scales better than single pooling.

After using the residual network as the feature extractor to extract the features, we need to further learn and process the extracted features to different sizes. These feature maps can be stacked into spatial pyramid feature maps. The pyramid feature map is subjected to different convolutions for feature learning and then resampled to restore the size of the input feature map. Then, these feature maps are combined with the input feature maps in a splicing manner. PPM can overcome the problems such as many parameters, difficult training, information loss, and overfitting and integrate features from four different pyramid scales. The gray module in PPM represents the feature map obtained after network training and continues to extract features with blocks having sizes of 1 × 1, 2 × 2, 3 × 3, and 6 × 6 pixels. We put these three grids on the feature map and can get 12 different blocks, and extract a feature from each block. Thus, 12 groups of features can be obtained. Obviously, the PPM can effectively combine features of different scales, including both deep-level high-level semantic features and low-level structural features. Accordingly, it can better learn and merge features.

**Figure 2:**
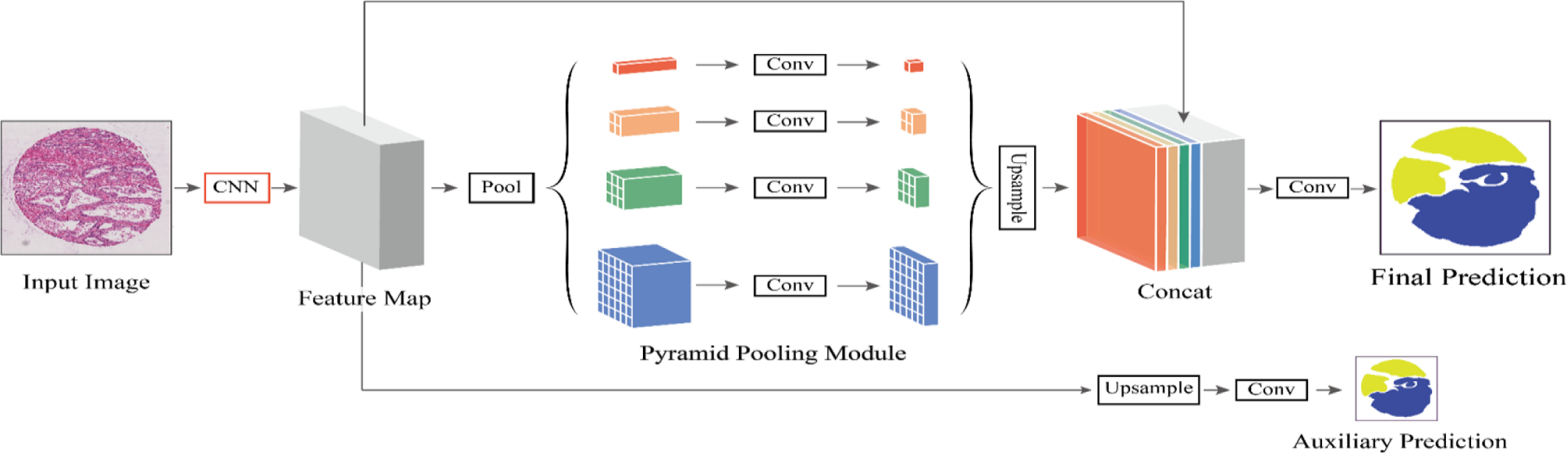
The flowchart of our proposed method. We define CNN as the basic feature extraction layer of our proposed network. By giving the original image, we first obtain the feature mapping of the last network convolution layer using CNN and then make an auxiliary prediction for the feature mapping by upsampling and concatenation layers. Next, we obtain the final feature representation with local and global information through different subregion representations in the PPM after upsampling and connection layers. Finally, these representations are fed into a convolution layer to get the final per-pixel prediction.

In **Figure 2**, different levels of output in the PPM contain different sizes of feature maps. The bold red highlight represents a single bin output. When the size of the pyramid layers set by the network is *N*, the corresponding global size decreases to 1*/N* of the initial size, which is sampled to the same size as the original feature map after low-dimensional feature mapping by bilinear interpolation. To better weigh the global feature weights, we add a 1 × 1 convolution layer after the pyramidal pooling layer. Finally, different levels of features are connected at different levels as the final global feature.

### Auxiliary Branch Network

This auxiliary branch network undergoes operations such as convolution and upsampling to output a prediction result. Note that the loss function of the backbone network is *L*_1_ and the loss function of the auxiliary branch network is *L*_2_, where the loss function *L* of the entire PSPNet network can then be expressed as:


(1) $$L=L_{1}+\alpha \cdot L_{2}$$

where α is a weight coefficient that balances the 0two loss functions, and both *L*_1_ and *L*_2_ are cross-entropy loss functions. Finally, we set α = 0.5.

Unlike the traditional backpropagation loss of its auxiliary blocking relays to the shallow layer of the network, we use two different loss mechanisms that can pass through all convolution layers for network calculation. The auxiliary loss can optimize the network learning without affecting the learning of the main branch. The combination of local and global information can effectively avoid information loss and make the diagnosis and prediction of diseases more reliable. During testing, we use the main branch with better optimization for final prediction. Specifically, we obtain the feature map by feeding the pathological slice image of prostate cancer into ResNet101. Subsequently, the feature map is fed into the PPM for multi-scale feature learning, and then the learned features pass through several convolution layers. Finally, we can get the final prediction result.

By constructing a feature pyramid to extract features of different sizes, each feature has rich image information, which improves the reliability of network feature extraction. The network model reveals the tradeoff between memory and accuracy and achieves good segmentation performance. Aiming at solving the problem of digital histopathological segmentation of prostate cancer, this method has high accuracy and robustness. Our method helps us get the champion of automatic prostate Gleason grading challenge 2019 which placed 1st on the MICCAI 2019 prostate segmentation benchmark, as well as the Vancouver Prostate Centre dataset.

## Experimental Setup and Results

### Dataset

**Figure 3:**
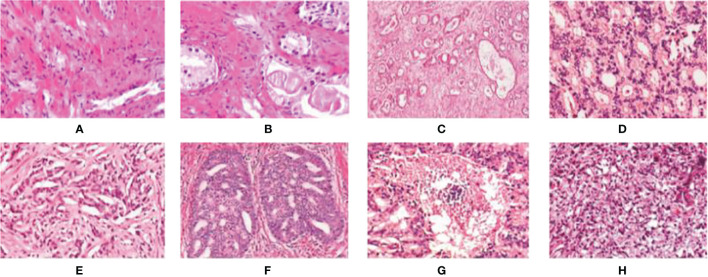
Examples of digital histopathology images for Prostate Gleason Grading. We list four different Gleason grade groups: **(A, B)** Benign; **(C, D)** Grade 3; **(E, F)** Grade4; and **(G, H)** Grade 5. It can be observed that each level has a variety of sizes, shapes, and irregular object boundaries. Gleason Grade 3 consists of well-formed and well-defined glands with varying sizes but which are smaller and tighter than non-cancerous prostatic tissue. While Gleason Grade 4 is associated with poorly formed glands, gland fusion is no longer separated by the matrix, even related to a pattern known as cribriform. Gleason Grade 5 includes the worst differentiated glands.

In **Figure 3**, we can see the differences at each gleason grade groups in detail. The histopathological data in this article are provided by the Vancouver Prostate Center, which are collected from different medical institutions and process tissue microarray blocks. It should be noted that the prostate tissue microarray is gained from patients with a suspicion of having prostate cancer. For the training dataset, the Gleason-level determination of the core image of each tissue microarray is associated with the most prevalent and second prevalent Gleason grading of expert annotations.

### Data Processing

The 2019 MICCAI Gleason Competition provides annotations from six experts, and the annotations provided by each expert are not necessarily complete. Fortunately, the annotation by all annotators completely covers all the data. Therefore, using these annotations becomes a primary challenge. All TMA core images are annotated as benign and Gleason patterns 3, 4, and 5. Six pathologists drew regions (closed contours) on pathological images and labeled each area with grades. Although all of the TMA core images are annotated in detail by pathologists, none of them is complete. There are even two images in the expert’s annotation without a corresponding image in the training set. However, one real tag is needed for network training; we need to merge the tags of six experts to enhance the robustness. Only four of the six pathologists label all the images, while the other two pathologists label only 191 and 92 images. To make better use of all expert annotations, we use the STAPLE algorithm to build the finally “ground truth label” *via* merging the annotations provided by multiple annotators.

### Implementation and Data Augmentation

To demonstrate the effectiveness of our method, we participate the MICCAI Gleason 2019 Challenge^1^. A total of 331 images (including 224 train images and 87 test images) are used from tissue microarray blocks. These images are manually annotated by professional dermatologists.

We use PyTorch for the distributed parallel training of algorithmic models on an Ubuntu high-performance graphic workstation. The learning rate decay strategy is $\eta =\eta_{0}{\left(1-\frac{n}{N}\right)}^{\beta }$ , where *η*_0_ = 0.002 is the initial learning rate, *n* is the current training rounds, *N* = 200 is the total training rounds, and *β* = 0.9.

Due to the large size of the prostate pathological slice image, it cannot be directly input to the network for learning. In this paper, we directly scale the short side of the input image to a size of 1,024 pixels and then randomly crop an 800 × 800 image patch as the input of the network. All samples are divided into five parts (each subset has an approximately equal number of samples). We repeat the entire process five times to avoid possible deviation of dataset partition during cross-validation. The final results are calculated by averaging five group results. To avoid network overfitting caused by insufficient data, we propose a data augmentation technique by randomly resizing and mirror training datasets.

### Evaluation Metrics

Performance metrics such as accuracy, mean, entropy, and standard deviation were used to evaluate performance. In this article, we evaluate the network performance through the evaluation metrics such as the distance similarity coefficient (DSC), Jaccard Index (JA), Hausdorff distance (HD), Cohen’s kappa coefficient, and F1 score.

The distance similarity coefficient (DSC) is defined as follows:


(2) $$\text{DSC}=\frac{2|A\cap B|}{\left|A|+|B\right|}$$

where │·│ represents a set of pixels, and *A* and *B* represent the real label and segmentation result, respectively. The Hausdorff distance (HD) is defined as follows:


(3) $$\text{HD }(X_{S},Y_{S})=\max(h(X_{S},Y_{S}),\;h(Y_{S},X_{S}))$$

where *X_S_* and *Y_S_* represent the point set of the real label and the segmentation result, respectively. *h*(*X_S_*, *Y_S_*) and *h*(*Y_S_*, *X_S_*) can be calculated as follows:


(4) $$h(X_{S},Y_{S})=\max_{x_{i}\in X_{S}}\min_{y_{j}\in Y_{S}}||x_{i}-y_{j}||$$

(5) $$h(Y_{S},X_{S})=\max_{y_{j}\in Y_{S}}\min_{x_{i}\in X_{S}}||y_{j}-x_{i}||$$

where ║·║ represents Euclidean distance. The average surface distance is another distance evaluation index. The lower the HD value, the better the network performance. Cohen’s kappa efficient *k* is defined as follows:


(6) $$\kappa =\frac{p_{0}-p_{e}}{1-p_{e}}$$

Where *p*_0_ is the relative scoring consistency of raters, and *p_e_* refers to the observed data used to calculate the hypothetical probability. If the scores of the two scores are the same, then *κ* = 1.


(7) $$\text{Precision}=\frac{\text{TP}}{\text{TP}+\text{FP}}$$

(8) $$\text{Recall}=\frac{\text{TP}}{\text{TP}+\text{FN}}$$

(9) $$\text{JA}=\frac{\text{TP}}{\text{TP}+\text{FN}+\text{FP}}$$

(10) $$F_{1}=2\frac{precision\times recall}{precision+recall}$$

where TN, TP, FN, and FP represent true negative, true positive, false negative, and false positive, respectively.

### 2019 MICCAI Automatic Prostate Gleason Grading Challenge

The 2019 MICCAI prostate grading challenge provides a unique dataset and strict evaluation conditions of Gleason grading for the challenging task. During the experiment, each region in the pathological section is mapped to the Gleason’s mode, and the low-level mode corresponds to the nearly normal prostate tumor. The differentiation level with the largest area is registered as the most important differentiation value, while the differentiation level with the second largest area is registered as the secondary differentiation value. The third largest or less than 5% minority is ignored. The prostate grading challenge includes two different tasks. One is the prediction of pixel-level Gleason grade, the other one is the prediction of core-level Gleason grade. Task 1 is regarded as a segmentation task, and we utilize PSPNet to accomplish this task. For task 2, we do not train a different network but provide a prediction of task 1 according to the Gleason grading system.

#### 1) Pixel-Level Gleason Grade Prediction

**Figure 4:**
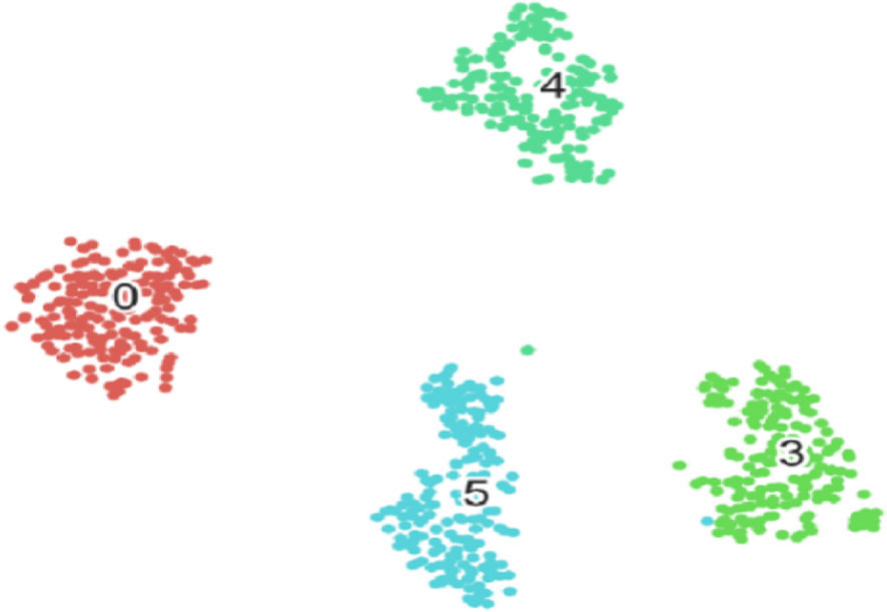
Our proposed method for T-SNE visualization with four-Gleason grading, where 0 represents benignity and 3, 4, and 5 represent Gleason grading 3, 4, and 5, respectively.

In the first task of this competition, the content is segmented into four pathological sections of different Gleason patterns. The evaluation metrics in this task provided by the competition-organizing committee are a combination of F1 scores and Cohen’s kappa coefficient. The coefficient is considered as an agreement to evaluate reliability and generally accepted to be a more robust measure rather than simple percent calculation. We use this combination score formula to calculate the final score on each test image. **Table 1** shows the predicted results of the pixel-level Gleason grade (note that the data in **Table 1** can be found on the official website of the competition). **Figure 4** shows T-SNE visualization with four-Gleason grading using our proposed method.

**Table 1:** Task 1: pixel-level Gleason grade prediction.

Rank	Team	Score
10	qq604395564	0.643761
9	jpviguerasguillen	0.649812
8	AlirezaFatemi	0.712537
7	XiaHua	0.716059
6	cvblab	0.757838
5	sdsy888	0.759776
4	zhangjingmri	0.778061
3	ternaus	0.789663
2	nitinsinghal	0.792585
1	Ours	0.845152

#### 2) Core-Level Gleason Grade Prediction

**Figure 5:**
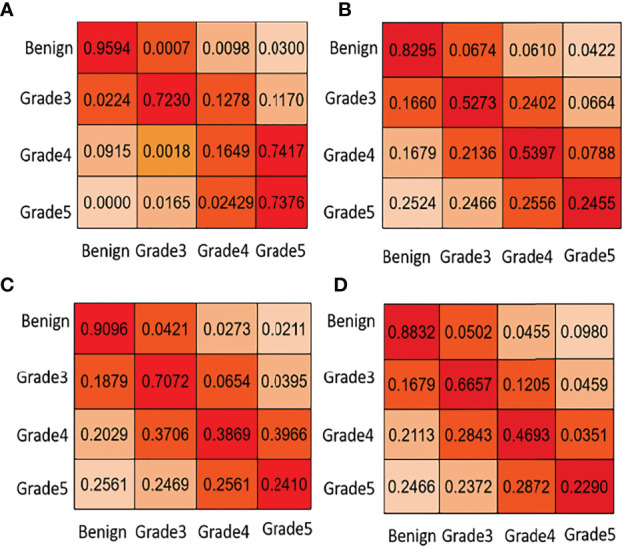
Task 2: core-level Gleason grade prediction. We list some confusion matrix results for different teams in this figure. **(A)** PSPNet (Our), **(B)** U-net++ (Ni), **(C)** U-net++ (Ternaus), **(D)** U-net (Zhang).

In the second task of this competition, the content is transformed from the segmentation of pathological sections to the classification of different Gleason patterns. To achieve automatic Gleason classification of prostate pathological images, we focus on not only segmentation but also the effect of network grading. We are interested in screening benign plaques from all biopsy pathological tissues. In addition, we focus more on distinguishing between high-grade cancers (Grades 4, 5) and low-grade cancers (Grade 3). Task 2 classifies and grades Gleason according to the results of task 1. Its classification effect is far more than that of the direct classification of pathological sections. Ignoring the background part of the image in the pixel-level Gleason grading result, we get the Gleason grading result with the largest proportion in the image. By comparing the predicted results with the direct calculation of the image-level Gleason grading results, we can analyze the image-level Gleason grading performance of each network. To verify the superiority of automatic grading of our network, we list the confusion matrix results of the top four contests. **Figure 5** lists the confusion matrix results for different teams. Note that the confusion matrix is derived from the whole training and test dataset on the Gleason grade group.

In **Figure 5**, the first line of the confusion matrix provided by group (a) shows that 95.94% of benign pixels are classified correctly; 0.07% of benign pixels are wrongly classified to grade 3, 0.98% to grade 4, and 3.00% to grade 5. From **Table 1** and **Figure 5**, we can see that our method consistently obtains good results in terms of the listed evaluation metrics and achieves the best segmentation results. Given the validity and reliability of the Gleason grading system in the detection and diagnosis of prostate cancer, our results are useful to the medical community in prostate cancer diagnosis.

### Comparison of Different Methods

For the training of the deep neural network, the amount of image information is associated with the size and number of input images. However, in the process of network training, the increase in network depth may bring additional optimization problems. According to the previous literature (38), FCN based on the residual network solves this problem *via* skipping connection in each block. We compare the four proposed different classical semantic segmentation networks, i.e., FCN (22), SegNet (39), U-Net (40), and DeepLabv3 (41), to verify the segmentation performance. The results are reported in **Table 2**.

**Table 2:** Results of different network models (boldface denotes best performance).

Model	DSC	kappa	JA	Score	HD
FCN (22)	0.784	0.753	0.728	0.717	0.312
U-Net (40)	0.812	0.774	0.742	0.757	0.266
SegNet (39)	0.833	0.788	0.751	0.732	0.242
DeepLabv3 (41)	0.859	0.814	0.821	0.798	0.213
PSPNet (ours)	**0.871**	**0.847**	**0.836**	**0.827**	**0.190**

Bold values represents the best performance.

To maintain consistency, we compare the three networks based on ResNet or with a network structure like ResNet in our prediction of pixel-level Gleason grade and core-level Gleason grades. During the experiment, we quantify Cohen’s quadratic kappa statistics to compare the consistency among pathologists, and the annotator consistency between models and pathologists. The comparison of pixel-level grade prediction of different methods is shown in **Table 3**.

**Table 3:** Pixel-level Gleason grade prediction of different methods.

Method	*κ*	*F*_macro_	*F*_micro_
FCN (22)	0.504 ± 0.128	0.605 ± 0.185	0.835 ± 0.126
DeepLabv3 (41)	0.438 ± 0.119	0.549 ± 0.188	0.773 ± 0.165
PSPNet (ours)	**0.524 ± 0.135**	**0.634 ± 0.198**	**0.839 ± 0.133**

Bold values represents the best performance.

As the doctors are concerned about the benignity and the malignancy of tumors at actual clinical practice, they are interested to make the distinction between high-risk cancer (Gleason patterns 4, 5) and low-risk cancer (Gleason pattern 3). Also, the classification results are based on high and low scores. Comparing the prediction results with the real labels, the core-level Gleason classification performance of each network is shown in **Table 4**. The comparative results of the proposed method with several recently proposed methods are reported in **Table 5**.

**Table 4:** Core-level Gleason grade prediction of different methods.

Method	Benign VS. malignant				Gleason 3 VS. Gleason 4 and 5			
	Accuracy	Precision	Sensitivity	Specificity	Accuracy	Precision	Sensitivity	Specificity
FCN (39)	0.925	0.963	0.954	0.714	0.813	0.904	0.801	0.835
DeepLabv3 (41)	0.902	0.944	0.944	0.600	0.729	0.840	0.734	0.718
PSPNet (ours)	**0.934**	**0.972**	**0.954**	**0.869**	**0.836**	**0.904**	**0.831**	**0.846**

Bold values represents the best performance.

**Table 5:** Comparison of the proposed method with several recently proposed methods.

Method	Benign VS. malignant			Gleason 3 VS. Gleason 4 and 5		
	Accuracy	Sensitivity	Specificity	Accuracy	Sensitivity	Specificity
Arvaniti et al. (42)	0.82	0.85	0.79	0.77	0.81	0.74
Nagpal et al. (43)	0.81	0.81	0.79	0.76	0.77	0.74
Davood et al. (44)	0.85	0.86	0.85	0.82	0.82	0.82
Ours	**0.94**	**0.96**	**0.87**	**0.84**	**0.83**	**0.85**

Bold values represents the best performance.

From **Table 3**, we can know that the PSPNet used in this study is superior to other comparative methods, which is consistent with the scores of six experts (calculated by Cohen’s kappa coefficient *k*) or the macro-average and micro-average F1 scores. On the test cohort, the minimum and maximum *k* of each of the six experts respectively scored by PSPNet are 0.23 and 0.62, while the minimum and maximum *k* between the six experts are 0.38 and 0.70. Therefore, the consistency of the PSPNet score between each expert and Gleason grade at the pixel level is within the consistency range of these six experts’ scores.

As is shown in **Tables 4**, **5**, the proposed PSPNet achieves the best performance with respect to various evaluation metrics for distinguishing benign from malignant. For distinguishing high and low score tumors, most performance metrics of the methods listed in the table are reduced, indicating that more difficulty existed to identify the high and low score tumors. However, PSPNet still acquires the best result, which further illustrates the stability and reliability of the method.

## Discussion

There may be numerous ways to improve this model, ranging from the overall architecture to the sampling of data. Despite the promising results of our model, it is not guaranteed that the application of this model in actual disease diagnosis leaves no room for errors. In this case, the tumor must be removed as far as possible without damaging other healthy tissue.

Regarding task 2 core level prediction, we have not directly calculated it. The final predicted results will be submitted to the evaluation platform, and the competition organizers will conduct experimental evaluation. From **Figure 5**, other participants have poor predictions on Gleason 5 and miss many high-scoring tumors, while the PSPNet method we use could detect Gleason 5 tumors well, but the discrimination between Gleason 3 and 4 is not good enough.

We also discuss the poor results of Gleason grade 4 with many experts and scholars and draw a conclusion that there is a certain randomness in the testing data selected in the experimental evaluation (Gleason grade 4 and Gleason grade 5 are very similar in shape), and the error rate of estimation is very high. However, pathologists believed that Gleason grade 4 and Gleason grade 5 are high-risk grades, which threatens the health and prognosis of patients. Although PSPNet achieves the lowest prediction accuracy of Gleason grade 4 at the core level, the automatic grading of prostate pathological images is not affected.

**Figure 6:**
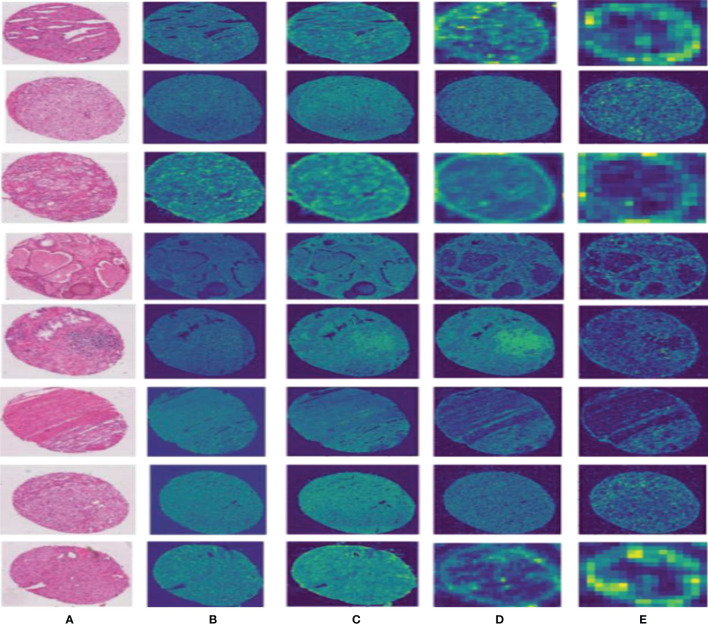
Results of the feature map visualization. Column **(A)** is the input image; columns **(B–E)** show that feature maps are obtained *via* four convolutional layers in PPM, respectively.

To effectively improve the diagnostic accuracy of the disease, we use a novel model to automatically grade digital prostate cancer histopathology. To prove the superiority of the proposed network, we extract feature maps from the residuals and visualize them, as shown in **Figure 6**; we can observe that our method effectively preserves the original input image information.

**Figure 7:**
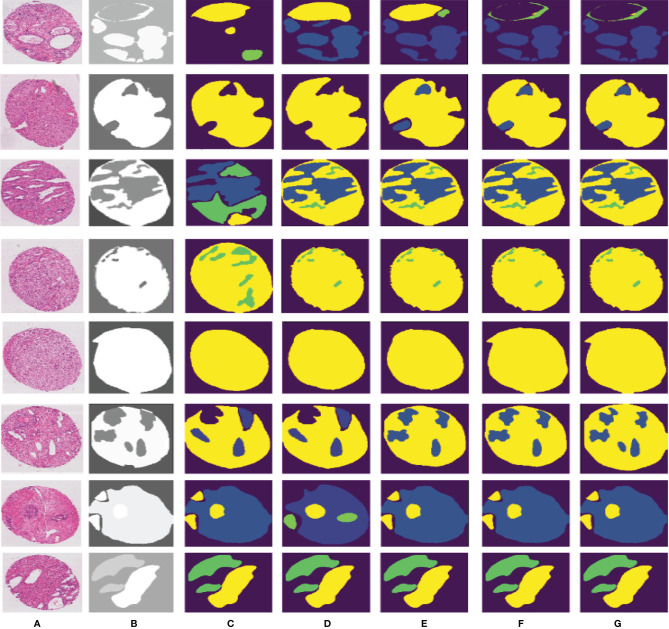
Different segmentation results from FCN, SegNet, U-Net, DeepLabv3, and our proposed method. Column **(A)** is the input image, column **(B)** is the final expert annotation used by the sample algorithm, and columns **(C–G)** indicate the final segmentation results of FCN, SegNet, U-Net, DeepLabv3, and PSPNet, respectively.

Compared to other teams, PSPNet performs the best among the submissions in MICCAI Automatic Prostate Gleason Grading Challenge 2019. All dynamic selections are automatically determined by PSPNet. The overall static design and dynamic selection are based on rules determined by our expertise in the field. These segmentation results of some challenging and representative samples are shown in **Figure 7**.

**Figure 8:**
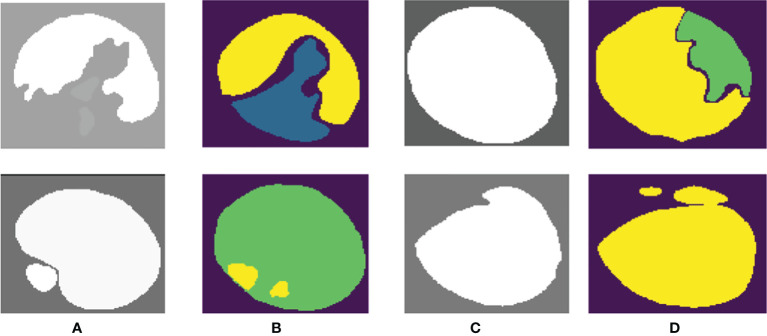
Poor-performing images using our method. Columns **(A, C)** are the “ground truth”, and columns **(B, D)** are the final segmentation result.

PSPNet can effectively learn multi-scale features to accurately classify prostate pathological images automatically. Even though this method has achieved good segmentation results, it still has some shortcomings. The main drawback of our method is that our training dataset is not enough for disease diagnosis for medical image analysis. Although the original data set has been increased 5-fold by data enhancement technology, we are still unable to guarantee its complete application to feature learning of deep neural networks. What is more, our method cannot segment some images well, as shown in **Figure 8**. We can observe that the three categories of Grade 3, Grade 4, and Grade 5 are easily confused.

Our proposed network can easily distinguish benign from malignant, but the differentiation of Gleason grades 3, 4, and 5 is insufficient. We consider that this may be related to the imbalance of the data distribution of each score in the dataset. In our training data, Gleason 5 has a small amount of data, so there are some errors in the results of network learning.

## Conclusion

In this paper, we propose a new automatic identification system for prostate biopsy tissues and use the STAPLE algorithm to synthesize different expert labels. The results based on cross validation show that our method achieves promising results in classification performance. The system could potentially improve the prognosis of prostate cancer and is highly consistent with the reference standard.

In the future work, some improvements could be taken into account from several sides. First, our approach puts particular emphasis on using image features as input to the network model. However, this handcrafted feature limits the richness of image structure information. To make the most of the power of the convolutional network model to acquire image features, fusing the hand-crafted features from original data and using more advanced network models to devise our framework could be tried. Second, we can bring other sophisticated factors into the proposed framework to potentially improve performance. Finally, we need to suggest to better integrate deep learning systems with the diagnosis processes of pathologists, and the impact of this artificial intelligence-based auxiliary method on the overall efficiency, accuracy, and prognosis of Gleason’s score in clinical practice.

## Data Availability Statement

Publicly available datasets were analyzed in this study. These data can be found in the following competition website: https://Gleason2019.grand-challenge.org/Home/.

## Author Contributions

YQ and YH wrote the programs, performed the data analysis, and drafted the manuscript. PK, HX, and XZ helped to check the contours. TW, JC, and BL guided the study and participated in the discussions and preparation of the manuscript. All authors read, discussed, and approved the final manuscript, and conceived the study design.

## Funding

This work was partly supported by National Natural Science Foundation of China (Nos.81771922, 62071309, 61801305, 62006160, 81971585, 62106153 and 61871274), National Natural Science Foundation of Guangdong Province (No.2019A1515111205), Shenzhen Key Basic Research Project (Nos. JCYJ20170818094109846, JCYJ20180507184647636, JCYJ20190808155618806, GJHZ20190822095414576, and JCYJ20190808145011259).

## Conflict of Interest

The authors declare that the research was conducted in the absence of any commercial or financial relationships that could be construed as a potential conflict of interest.

## Publisher’s Note

All claims expressed in this article are solely those of the authors and do not necessarily represent those of their affiliated organizations, or those of the publisher, the editors and the reviewers. Any product that may be evaluated in this article, or claim that may be made by its manufacturer, is not guaranteed or endorsed by the publisher.

## Footnotes

1 Competition website: https://Gleason2019.grand-challenge.org/Home/
